# Alcohol’s Effects on Adolescents

**Published:** 2002

**Authors:** Linda Patia Spear

**Affiliations:** Linda Patia Spear, Ph.D., is a Distinguished Professor and chairperson in the Department of Psychology at the Center for Developmental Psychobiology, Binghamton University, Binghamton, New York

During adolescence, many people begin to experiment with alcohol, yet relatively little is known about alcohol’s effects on this critical stage of development. We do know that early initiation of alcohol use remains one of the most powerful predictors of later alcohol abuse (Grant 1998). We also know that during adolescence changes occur in the regions of the brain involved in modulating drug reinforcement, so it cannot be assumed that factors precipitating alcohol use or abuse are the same in adolescence as in adulthood. Rapidly changing body systems often are particularly vulnerable to disruption, and hence long-term consequences may result from alcohol exposure during this time of accelerated neural and endocrine system maturation (Spear 2000*a*). For all of these reasons, adolescence is a critical stage of development, and additional research is warranted into the effects of drinking during this important transition period. This sidebar briefly reviews findings on how alcohol affects adolescents, with a special emphasis on the impact of alcohol on neural and endocrine development. Though the research in this area is scarce, gender-specific effects are highlighted whenever possible.

## Epidemiology of Drinking Among Adolescents

Results from national surveys of adolescents and young adults show that alcohol use is prevalent among both young men and women. The prevalence of drinking and binge drinking (consuming five or more drinks on a single occasion in the previous 2 weeks) is higher among male students relative to their female peers, but data from the Monitoring the Future Survey (MFS) (Johnston et al. 2002)—a nationally representative sample of 8th, 10th, and 12th graders—show that the gender gap is closing. For example, in 2001, 36 percent of 12th grade males reported binge drinking, compared with 24 percent of their female counterparts (a 12-percentage-point difference). However, in 1975 there was a 23-percentage-point difference between rates of male and female binge drinking (Johnston et al. 2002). Among females, 20.6 percent of 8th graders and 45.1 percent of 12th graders reported using alcohol in the 30 days prior to the survey (i.e., 30-day prevalence); of those 8th grade females, more than half reported binge drinking.

## Early Initiation of Alcohol Use

This early alcohol use may have potentially long-lasting consequences. Early onset of alcohol or other drug use is one of the strongest predictors of later alcohol dependence (Grant 1998). Although young men are significantly more likely than young women to report using alcohol before age 13 (34.2 percent versus 24.2 percent) (Grunbaum et al. 2002), survey data suggest that, over time, the age of initiation to alcohol use among young women has decreased. For example, in 1975, 42 percent of female high school seniors reported first using alcohol before 10th grade, compared with 53 percent in 1993 (the last year for which the specific question was asked) (Johnston et al. 2001).

Two possible explanations exist to describe the relationship between early alcohol use and later dependence. First, exposure to alcohol or other drugs during adolescence may alter critical ongoing processes of brain development that occur at that time, increasing the likelihood of problems with alcohol later in life. Indeed, heavy drinking during early and mid-adolescence has been found to be associated with memory problems and other neuropsychological deficits, although the causality of this relationship has yet to be determined (Brown et al. 2000). Another interpretation for the early exposure effect is that early use of alcohol or other drugs might simply serve as a marker, not a precursor, for a later abuse disorder. For instance, a preteen’s tendency to seek out new experiences (i.e., high novelty-seeking behavior) was found to be predictive of alcohol abuse at age 27 (Cloninger et al. 1988). Strong novelty-seeking behavior is one of a number of traits that have been linked to early initiation of alcohol and other drug use (Baumrind 1987).

These two views on the significance of the early exposure effect are not necessarily mutually exclusive. For example, adolescents with conduct disorder are at higher risk for early as well as later alcohol and other drug use. Yet people with conduct disorder who begin to drink at an early age have a particularly high risk for problems with alcohol and other drugs later in life (Robins and McEvoy 1990).

## Neural and Endocrine Development

Striking physical changes occur in the brain during adolescence, including the maturation of new brain constituents (such as the formation of additional connections between nerve cells) as well as a prominent loss (or pruning) of some existing connections. Adolescence-associated changes in the brain’s dopamine (DA) system may affect the way this important neural messenger communicates with the prefrontal cortex and limbic brain regions (i.e., the so-called mesocorticolimbic DA system). Changes in these systems may have a profound effect on adolescent behavior and psychological functioning (Spear 2000*b*). It is possible that features of the adolescent brain may predispose young people to behave in ways that place them at particular risk for trying alcohol or other drugs. In rats, the DA system has been implicated in novelty seeking (Dellu et al. 1997) and has been identified as part of a brain cell circuit involved in assigning value (i.e., “incentive salience”) to stimuli, including alcohol, and translating the decision to use alcohol into action (Kalivas et al. 1993).

Adolescence also is the time during which changes in hormone patterns begin to emerge. Sex differences in behavior appear, orchestrated in part by the rapid changes in these pubertal hormones (for more information, see the article in this issue by Emanuele and colleagues, pp. 274–281). Surprisingly, though, puberty-related increases in reproductive hormones have not been associated in any simple way with other characteristic behavioral features of adolescence (Susman et al. 1987). Instead, the unique behavioral features of adolescence—such as a greater emphasis on peer interactions, increased novelty seeking, and other reckless behavior (Arnett 1992; Spear 2000*b*)—may be driven largely by maturational changes in the nervous system, as reviewed below.

During adolescence, the prefrontal cortex, a region thought to be involved in various goal-directed behaviors (e.g., rule learning, working memory, and spatial learning) and in emotional processing (particularly of unpleasant stimuli) undergoes substantial remodeling. For example, as demonstrated in nonhuman primates, the input from two key chemicals (i.e., neurotransmitters) involved in brain cell communication—the excitatory neurotransmitter glutamate and the inhibitory neurotransmitter gamma-aminobutyric acid (GABA)—is reduced during adolescence, while input from another neurotransmitter, DA, peaks in the prefrontal cortex during adolescence (Lewis 1997). Another region that undergoes developmental adjustment is the amygdala (Yurgelun-Todd 1998), a complex grouping of brain cells that, among other things, is thought to be involved in a person’s emotional reactions and in coordinating the body’s response to stress.

In research on another brain region, the hippocampus, which is important for learning and memory, DeBellis and colleagues (2000) used magnetic resonance imaging to evaluate the volume of this region in alcohol-abusing or alcohol-dependent adolescents (average age 17). The researchers found that hippocampal volumes were significantly smaller in the adolescents with alcohol use problems, compared with control subjects. Older age of onset of the alcohol use disorder and shorter duration of the disorder were associated with larger hippocampal volume. Although studies show that alcohol use affects neurocognitive function in adult women and men equally, female study participants’ shorter drinking histories suggest that they may be more sensitive to alcohol’s effects (Glenn et al. 1988; Nixon 1994). In addition, limited research suggests that women may be more susceptible than men to alcohol-related brain shrinkage (Hommer et al. 1996*a*,* b*).

## Responses to Stress

Gender differences in the body’s hormonal response to stress also begin to emerge late in adolescence. For example, compared with males, prepubescent female rats show elevated levels of corticosterone (analogous to cortisol in humans)—a key stress hormone (Ramaley and Olson 1974; Cirulli et al. 1996).

In addition, many of the same neural systems known to undergo developmental changes during adolescence are activated by stress, including DA projections to the pre-frontal cortex as well as to mesolimbic brain regions (Abercrombie et al. 1989)—areas thought to be critical in modulating the pleasurable response that follows alcohol use (Koob 1992). In studies with rats, important docking molecules (i.e., receptors) for the stress hormone corticosterone have been identified on DA cell bodies in the ventral tegmental area and the substantia nigra as well as in DA terminal regions, including the nucleus accumbens and the prefrontal cortex (Ahima and Harlan 1990; Cintra et al. 1994). Increases in corticosterone may play a critical role in activating DA transmission, as evidenced by the fact that, in rodents, DA levels in the nucleus accumbens (Piazza et al. 1996) and prefrontal cortex (Imperato et al. 1989) increase with corticosterone treatment and decrease with removal of the adrenals (the area where corticosterone is produced). In a similar fashion, adrenalectomy or pharmacologically induced blockade of stress-hormone synthesis suppresses alcohol consumption in laboratory animals (Fahlke et al. 1994).

The results of this basic research suggest that stress-induced increases in stress hormones may interact with mesocorticolimbic brain regions to facilitate alcohol use behavior. Further research into the effects of stress on the development of alcohol problems is crucial. Investigations of stress effects in adolescents will be especially important given the dramatic changes taking place in the brain during that time.

Likewise, further examination of how stress, anxiety, and depression interact in this age group is important. Adolescence often is characterized as an emotionally stormy period. Though most children navigate this transitional period without serious problems, about one-third to one-half of adolescents report significant depressed mood or affective disturbances that could be described as “inner turmoil” or “feeling miserable” (Compas et al. 1995; Rutter et al. 1976). Adolescents also tend to show greater extremes in mood than adults (for a review, see Larson and Richards 1994; Arnett 1999); in addition to this emotional volatility, anxiety and self-consciousness also appear to peak at this time (see Buchanan et al. 1992).

Pubertal maturation in girls is associated with emotional difficulties, depression, and problems with self-image, as well as an increase in risk-taking behaviors (for a review, see Steinberg and Belsky 1996). During early adolescence, girls may be especially vulnerable to stress, perceiving events to be more stressful at that time than at any other (Ge et al. 1994; Wagner and Compas 1990; also see Vik and Brown 1998 for further discussion of gender differences in perceived stressfulness during adolescence).

This anxiety and stress may play an important part in adolescents’ initiation of alcohol or other drug use (Pohorecky 1991; Wagner 1993). In her review of the literature on stress effects on alcohol consumption in humans, Pohorecky (1991) found that stress clearly influences alcohol consumption in adolescence, but not necessarily in adults. Indeed, the level of perceived stress was found to be the most powerful predictor of adolescent alcohol and other drug use, after peer substance use (Wagner 1993).

Researchers need more information about the hormonal, behavioral, and neural interactions that take place in response to stress during adolescence. Understanding why young people use alcohol to cope with stress within a developmental timeframe also is important. The relationship between stress and adult drinking may be far different from the relationship between these variables in adolescence, the time when most people begin drinking.

## Tolerance and Sensitivity to Alcohol’s Effects

Evidence suggests that alcohol may affect adolescents differently than adults. Studies using animals have shown that, compared with other age groups, adolescents do not experience the same degree of incoordination and sleepiness when drinking alcohol as do adults (that is, they are relatively resistant to the motor-impairing and sedative effects of alcohol) (Silveri and Spear 1998). Adolescents do, however, appear to be more sensitive to alcohol-induced disruptions in spatial memory (Markwiese et al. 1998). Research is needed to determine when young people in this age group are most susceptible to alcohol’s effects, what mechanisms underlie this differential age responsiveness, and whether female adolescents differ from males in alcohol sensitivity at this critical time. Understanding tolerance and sensitization is particularly important given that research suggests that a less intense reaction to alcohol may increase the likelihood that a person will drink more heavily and more often, setting the stage for the development of alcohol problems (Schuckit 1995).

## Conclusion

Research on alcohol’s effects on the developing adolescent is still in its infancy, despite the fact that this is the time during which many people begin drinking. There is evidence that people who begin drinking at an early age may have problems with alcohol later in life. Research also has shown that adolescence is a time when remarkable changes are taking place in the brain. Just how alcohol use impacts this development or whether these developmental changes influence alcohol use is unknown.

It also is unclear how gender differences may influence the way that alcohol affects the developing adolescent brain and other body systems. Researchers have shown that chronic alcohol consumption can disrupt developmental changes in hormones associated with puberty in both males (Cicero et al. 1990) and females (Dees et al. 1990). It also is clear that gender influences the perception of stress, a factor that has been shown to lead to higher rates of alcohol use among this age group. Just how these endocrine-related changes influence alcohol use is not fully understood.

Most importantly, future research efforts must examine why early exposure to alcohol is apparently associated with considerably more adverse consequences than later use, and why this age group seems at particular risk for alcohol’s deleterious effects.
